# LLIN evaluation in Uganda project (LLINEUP): The fabric integrity, chemical content and bioefficacy of long-lasting insecticidal nets treated with and without piperonyl butoxide across two years of operational use in Uganda

**DOI:** 10.1016/j.crpvbd.2022.100092

**Published:** 2022-05-25

**Authors:** Frank Mechan, Agaba Katureebe, Violet Tuhaise, Martin Mugote, Ambrose Oruni, Ismail Onyige, Kawesa Bumali, Jonathan Thornton, Kilama Maxwell, Mary Kyohere, Moses R. Kamya, Peter Mutungi, Simon P. Kigozi, Adoke Yeka, Jimmy Opigo, Catherine Maiteki-Sebuguzi, Samuel Gonahasa, Janet Hemingway, Grant Dorsey, Lisa J. Reimer, Sarah G. Staedke, Martin J. Donnelly, Amy Lynd

**Affiliations:** a Department of Vector Biology, Liverpool School of Tropical Medicine, Liverpool, UK; b Infectious Diseases Research Collaboration, Uganda; c Department of Medicine, Makerere University, Kampala, Uganda; d Makerere University - Johns Hopkins University (MUJHU) Research Collaboration, Kampala, Uganda; e National Malaria Control Division, Ministry of Health, Kampala, Uganda; f Department of Medicine, University of California, San Francisco, USA; g Department of Clinical Research, London School of Hygiene & Tropical Medicine, London, UK

**Keywords:** LLIN, Malaria, Durability, Insecticide, Bioefficacy, Piperonyl butoxide

## Abstract

Long-lasting insecticidal nets (LLINs) supplemented with the synergist piperonyl butoxide have been developed in response to growing pyrethroid resistance; however, their durability in the field remains poorly described. A pragmatic cluster-randomised trial was embedded into Ugandaʼs 2017–2018 LLIN distribution to compare the durability of LLINs with and without PBO. A total of 104 clusters (health sub-districts) were included with each receiving one of four LLIN products, two with pyrethroid + PBO (Olyset Plus and PermaNet 3.0) and two pyrethroid-only (Olyset Net and PermaNet 2.0). Nets were sampled at baseline, 12 and 25 months post-distribution to assess physical condition, chemical content, and bioefficacy. Physical condition was quantified using proportionate Hole Index and chemical content measured using high-performance liquid chromatography. Bioefficacy was assessed with three-minute World Health Organisation (WHO) Cone and Wireball assays using pyrethroid-resistant *Anopheles gambiae*, with 1-h knockdown and 24-h mortality recorded. There was no difference in physical durability between LLIN products assessed (*P* = 0.644). The pyrethroid content of all products remained relatively stable across time-points but PBO content declined by 55% (*P* < 0.001) and 58% (*P* < 0.001) for Olyset Plus and PermaNet 3.0 respectively. Both PBO LLINs were highly effective against pyrethroid-resistant mosquitoes when new, knocking down all mosquitoes. However, bioefficacy declined over time with Olyset Plus knocking down 45.72% (95% CI: 22.84–68.62%, *P* = 0.021) and Permanent 3.0 knocking down 78.57% (95% CI: 63.57–93.58%, *P* < 0.001) after 25 months. Here we demonstrate that both Olyset Plus and PermaNet 3.0 are as durable as their pyrethroid-only equivalents and had superior bioefficacy against pyrethroid-resistant *An. gambiae*. However, the superiority of PBO-LLINs decreased with operational use, correlating with a reduction in total PBO content. This decline in bioefficacy after just two years is concerning and there is an urgent need to assess the durability of PBO LLINs in other settings.

## Highlights

• Olyset Plus and PermaNet 3.0 nets effective against pyrethroid-resistant *Anopheles gambiae* when new.
• Killing effect of both nets halved after two years.
• Nets in ‘traditional’ thatched-roof housing at high risk of damage.

## Graphical abstract

**Figure:**
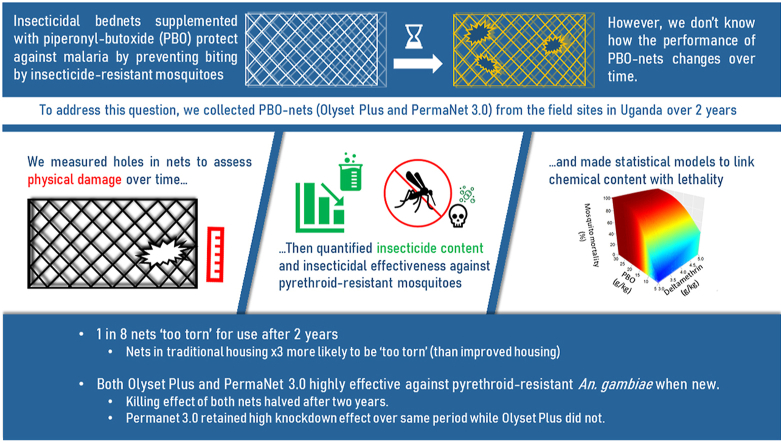

## 1 Introduction

Long-lasting insecticidal nets (LLINs) are the cornerstone of global malaria control strategies, forming a physical and chemical barrier against the bites of *Anopheles* mosquitoes (Bhatt et al., 2015; Churcher et al., 2016; Pryce et al., 2018). Progress in reducing malaria burden in sub-Saharan Africa achieved in the first decade of the 21st century has been attributed, in large part, to mass distribution of LLINs (Bhatt et al., 2015). LLINs are intended to maintain an effective level of protection for at least three years, with the expectation that distributions will take place at two-to-three-year intervals (WHO, 2013a, 2016). However, recent studies suggest that the lifespan of LLINs may be less than three years (Gnanguenon et al., 2014; Toé et al., 2019; Lorenz et al., 2020). To ensure the continued success of malaria control efforts, National Malaria Control Programmes (NMCPs) must identify LLIN products that demonstrate durability within the socio-economic and environmental context of their country.

The World Health Organisation (WHO) currently recommends the use of pyrethroid and pyrrole insecticides on LLINs (WHO, 2017b); however, the effectiveness of LLINs is threatened by widespread pyrethroid resistance (Ranson & Lissenden, 2016; Churcher et al., 2016; Hemingway et al., 2016). The development of target site alterations and metabolic resistance enables mosquitoes to better tolerate insecticide exposure, increasing the probability they will obtain a blood meal and survive the encounter (Irish et al., 2008; Asidi et al., 2012; Strode et al., 2014). While there is evidence that pyrethroid LLINs retain some protective effect against resistant mosquito populations (Alout et al., 2016; Viana et al., 2016), the threat of resistance has incentivised the development of new classes of LLIN. Due to the limited alternatives to pyrethroids, initial efforts to maintain the impact of LLINs have focused on secondary compounds that restore the susceptibility of pyrethroid-resistant mosquitoes. Piperonyl butoxide (PBO) is a synergist that inhibits the cytochrome P450 enzymes within the mosquito which detoxify insecticides (Darriet & Chandre, 2011). In 2017, the WHO provided an interim endorsement of use of pyrethroid LLINs containing PBO in areas of moderate pyrethroid resistance (WHO, 2017a) and a 2021 Cochrane review concluded that PBO-LLINs were associated with a reduction in parasite prevalence in areas of moderate-high pyrethroid resistance compared to pyrethroid-only nets (Gleave et al., 2021). However, the same review emphasised that evidence of the durability of these PBO-LLINs under operational conditions is lacking.

LLINs are known to lose insecticide content during routine use (WHO, 2013b). As nets are handled and washed, the insecticide at the surface is depleted then gradually regenerated by a reservoir within the fibres (Gimnig et al., 2005). Pyrethroid LLINs are designed with sufficient insecticide reserves to continue regenerating for at least three years, with the expectation they will be replaced before this time (WHO, 2013a). Currently, WHO LLIN durability guidelines quantify performance against objective bioefficacy benchmarks to assess if a three-year operational lifespan is achieved (WHO, 2011, 2013a), yet there is emerging evidence to suggest that bioefficacy varies substantially between products and may fall below defined efficacy thresholds within three years (Gnanguenon et al., 2014; Toé et al., 2019; Lorenz et al., 2020).

In Uganda, the country with the highest malaria burden in East Africa, progress in controlling transmission has faltered (Lynd et al., 2019). The declining efficacy of conventional control strategies coincides with emerging evidence of both high levels of knockdown resistance (*kdr*) and metabolic resistance in mosquito populations throughout the country (Lynd et al., 2019; Njoroge et al., 2021). As part of a commitment to achieve universal coverage of LLINs, the Ugandan Ministry of Health initiated a mass distribution of LLINs and PBO LLINs in 2017. A randomised control trial was embedded within this distribution programme to evaluate the impact of LLINs with and without PBO (Staedke et al., 2019). From this, it was demonstrated that PBO-LLINs reduce parasite prevalence in children aged 2–10 years-old and vector density more effectively than conventional LLINs for at least 25 months (Staedke et al., 2020; Gleave et al., 2021). The present study was conducted as part of the same trial to evaluate the durability of the PBO-LLINs. Here the physical integrity, chemical integrity, and bioefficacy of two PBO-LLIN products are assessed in comparison with their pyrethroid-only equivalents at 12 and 25 months post-distribution.

## 2 Materials and methods

### 2.1 Study site

The trial protocol for this study has been published (Staedke et al., 2019)^.^ A total of 104 clusters (health sub-districts, HSDs) in eastern and western Uganda were randomly assigned to receive one of four LLIN products, including two LLINs with PBO (PermaNet 3.0 and Olyset Plus) and two LLINs without PBO (PermaNet 2.0 and Olyset Net). Cross-sectional community surveys were carried out in 50 households per cluster (5200 households per survey) to confirm presence of the expected LLIN product from the distribution and entomological surveillance undertaken in 10 households per cluster. Efficacy data from this study have been published previously (Staedke et al., 2020). In the present study, we quantify the chemical and physical integrity of 400 LLINs, 97–100 nets of each type (Supplementary Table S1), withdrawn from households after 12 months and 25 months (total of 800 nets). These nets were assessed alongside unused nets of the same LLIN products.

### 2.2 LLIN description

Four LLIN products were distributed and assessed in this study: Olyset Net treated with permethrin; PermaNet 2.0 treated with deltamethrin; Olyset Plus treated with permethrin and PBO; and PermaNet 3.0 treated with deltamethrin and incorporating PBO on the top surface of the net only. All nets were 180 cm long × 170 cm wide × 170 cm high; the chemical and fabric specifications of each LLIN product are shown in Table 1.

**Table 1:** Specifications of LLIN products assessed in study. The target dose was defined as the amount of chemical per kg of fabric.

Product name	Manufacturer	Fabric type	Active ingredient target dose (w/manufacturing tolerance)
Olyset Net	Sumitomo Chemical Ltd.	Polyethylene (150 denier)	Permethrin: 20 ± 5.0 g/kg
Olyset Plus	Sumitomo Chemical Ltd.	Polyethylene (150 denier)	Permethrin: 20 ± 5.0 g/kg PBO: 10 ± 2.5 g/kg
PermaNet 2.0	Vestergaard Frandsen	Polyester (100 denier)	Deltamethrin: 1.4 ± 0.35 g/kg
PermaNet 3.0	Vestergaard Frandsen	roof: Polyethylene (100 denier); sides: Polyester (75 denier)	Deltamethrin: 4.0 ± 1.0 g/kg (roof); 2.8 ± 0.525 g/kg (sides) PBO: 25 ± 2.5 g/kg (roof)

### 2.3 Field collections

Net sampling was performed at baseline, 12 months, and 25 months post-distribution. At baseline, a total of 20 nets were retained (5 of each LLIN product) from the LLINs that were to be distributed during the campaign to be used as baseline samples. Post-distribution, at 12 and 25 months, 100 LLINs of each type were collected from houses enrolled in the community survey (across the 104 clusters). This sample size was a pragmatic decision based on available human capacity and estimated processing time, and on availability of replacement nets.

Nets were sampled and exchanged for a new net of the same type. Nets were identified as part of the study by a unique ID number (net ID) attached to each net. If no study net was found at the selected household or the net was an unexpected type, then the next household on the reserve list was sampled instead. No more than one net per household was sampled. Information on the construction of the dwelling was recorded, with the household categorised as ‘improved’ if it had both brick walls and an iron roof. Otherwise, the dwelling was categorised as ‘traditional’.

On collection, sampled nets were labelled and placed individually in zip-lock bags. All sampled nets were transported to the project office in Bugembe, Jinja, Uganda, for physical assessment and processing. After physical measurements were recorded, seven 30 × 30 cm pieces were cut from each net (one from centre of each side panel and three from the top) and samples sent to the Liverpool School of Tropical Medicine (Liverpool, UK) for chemical and bioefficacy assessment.

### 2.4 Physical integrity

To assess the physical integrity of the net fabric, nets were placed over a metal frame measuring W160 × L180 × H170 cm and any holes > 0.5 cm recorded (Lorenz et al., 2014). The size of a hole was defined by its length (the longest dimension) and width (measurement perpendicular to length measurement). Holes smaller than 0.5 cm (in length or width) and holes that had been repaired were noted but not included in the final dataset. Hole size was calculated using the formula for an ellipse (area = π × length × width). The total area of damage on a net was summed and used to categorise the net within the WHO proportionate Hole Index (pHI) categories: ‘good’ (0–64 cm^2^), ‘damaged’ (65–642 cm^2^); or ‘too torn’ (643 cm^2^+) (WHO, 2013b). Additionally, the proportion of nets of each LLIN product with at least one hole was calculated for each time-point.

Following physical integrity testing, two 30 × 30 cm square net pieces were sampled from the top of each LLIN for bioefficacy and chemical assessment. The rationale for using pieces cut from the top for chemical and bioefficacy testing was to allow fair comparison with PermaNet 3.0 which has PBO on the roof only, as well as literature indicating that *Anopheles gambiae* (*s.l.*) activity around an occupied bednet is focussed primarily on the top surface (Lynd & McCall, 2013; Sutcliffe & Yin, 2014, 2021). The samples were wrapped in aluminium foil and stored at room temperature prior to use in WHO cone bioassays. Samples were subsequently stored at 4 °C until chemical content and bioefficacy was assessed.

### 2.5 Chemical integrity

To quantify the content of active ingredients, chemical analysis was performed using high-performance liquid chromatography (HPLC) after extraction in 10% 1-propanol in heptane. A total of 30 nets of each LLIN type were analysed at each time-point, with two samples taken from each net.

The HPLC analysis was performed on an Agilent 1100 Series machine (Aglient, California, USA) at a wavelength of 226 nm, using a modification of the methods published by Ngufor et al. (2022). Quantities of permethrin, deltamethrin and piperonyl butoxide were calculated by comparison to standard curves of each compound (PESTANAL®, analytical standard, Sigma-Aldrich, Missouri, USA) and corrected against internal standard dicyclohexyl phthalate (DCP). HPLC data were analysed using OpenLAB software v2.1 (Aglient, California, USA).

### 2.6 WHO cone bioassays

To assess bioefficacy, WHO cone bioassays were performed using the protocol outlined in the WHO durability monitoring guidelines (WHO, 2011, 2013a).

Bioefficacy testing was performed on the same nets assessed for chemical content. The two pieces from each net were each tested in duplicate, thus a total of four cone exposures were performed per net. Cone bioassay design followed the WHO protocol, with the testing board angled at 45° (WHO, 2011; Owusu & Müller, 2016). Ambient conditions in the testing room were targeted to a temperature of 27 ± 2 °C and a relative humidity of 80 ± 10%. All mosquitoes used were 3–5-day-old unfed females, reared in temperature and humidity-controlled insectaries. Each exposure lasted 3 minutes, with 7 mosquitoes per cone. Thus, 24 mosquitoes were used in each cone exposure assay per net piece for each mosquito strain.

Two different mosquito strains were used in the cone bioassays: ‘Kisumu’ and ‘Busia’. ‘Kisumu’ is a pyrethroid-susceptible strain of *An. gambiae* collected in 1975 from what is now Kisumu County (formerly Kisumu District), in western Kenya. ‘Busia’ is a strain established in November 2018 from mosquitoes collected in Busia, eastern Uganda, by Ambrose Oruni. This strain has been previously characterised as possessing resistance to pyrethroids through both target site alterations (*Vgsc*-1014S) and metabolic resistance mechanisms (*Cyp4j5*, *Cyp6aa1* and *Coeae1d*) (Lynd et al., 2019; Njoroge et al., 2021)^.^ WHO tube assays with standard discriminating doses indicate ‘Busia’ is more resistant to permethrin than deltamethrin (Supplementary Fig. S1).

WHO bioefficacy criteria are defined as the proportion of nets that achieve either 80% mortality or 95% knockdown against pyrethroid-susceptible *An. gambiae* (*s.s.*) mosquitoes. An LLIN product was considered to have passed if 80% of nets met these criteria at all time-points up to 24 months. Chemical and physical integrity data are not included in bioefficacy criteria.

### 2.7 WHO wireball assays

Given previous literature indicating that WHO cone bioassays are insufficient to assess the bioefficacy of LLIN products containing insecticides with high contact irritancy (WHO, 2006, 2011; Okumu et al., 2012), such as permethrin, supplemental WHO wireball assays were performed on the same samples used in the WHO cone bioassays. The purpose of this secondary testing was to assess bioefficacy under conditions where there were no surfaces on which the mosquito could rest to avoid contact (such as the cone itself in the WHO cone assay). While the WHO Tunnel test is recommended as a secondary assay for assessing nets with high contact irritancy, the present study could not undertake this technique due to the ethical issues surrounding the use of smalls mammals as bait.

In the WHO wireball method, the net to be tested is affixed around a wire cube measuring 15 × 15 × 15 cm (WHO, 2006). As in the cone bioassay, seven 3–5-day-old females were released into the wireball for three minutes then assessed for 1 h knockdown and 24 h mortality.

### 2.8 Data analysis

Data analyses were conducted using R (version 3.6.0), all graphs were produced using the *ggplot2* package (version 3.2.1). Associations between outcomes and variables of interest were quantified using generalized linear mixed models (GLMMs) using the *lme4* package (version 1.1-21). To account for unexplained variation between separate pieces from individual nets and between clusters, the net ID (a unique identifier for each net distributed) and HSD number were each included in the models as a random effect. The model selection process used stepwise regression, working backwards from a maximally complex model to produce the most parsimonious fit. Variables that did not significantly increase explanatory power, as indicated by log-likelihood ratio tests (LRTs) (*lmtest* package, version 0.9-37), were excluded from the final model. All possible interactions between variables were considered in the model selection process; for succinctness, only significant interactions are presented. The *P*-values reported are the output of these LRTs. Pairwise comparisons between levels within a categorical variable were performed using least square means with the *lsmeans* package (version 2.30-0).

To quantify the relationship between chemical integrity and bioefficacy, the HPLC outputs for each net were combined with their corresponding WHO cone assay or WHO wireball assay mortality data (for PermaNet 3.0 and Olyset Plus, respectively). A GLMM was then fit separately to the PermaNet 3.0 and Olyset Plus data, with pyrethroid content and PBO content each fit as a fixed effect. Model selection and *P*-value reporting was performed as above. The 3D plots were produced using the *plot3D* package (version 1.4).

## 3 Results

### 3.1 Physical integrity

#### 3.1.1 Proportion of nets in each pHI category

**Fig. 1:**
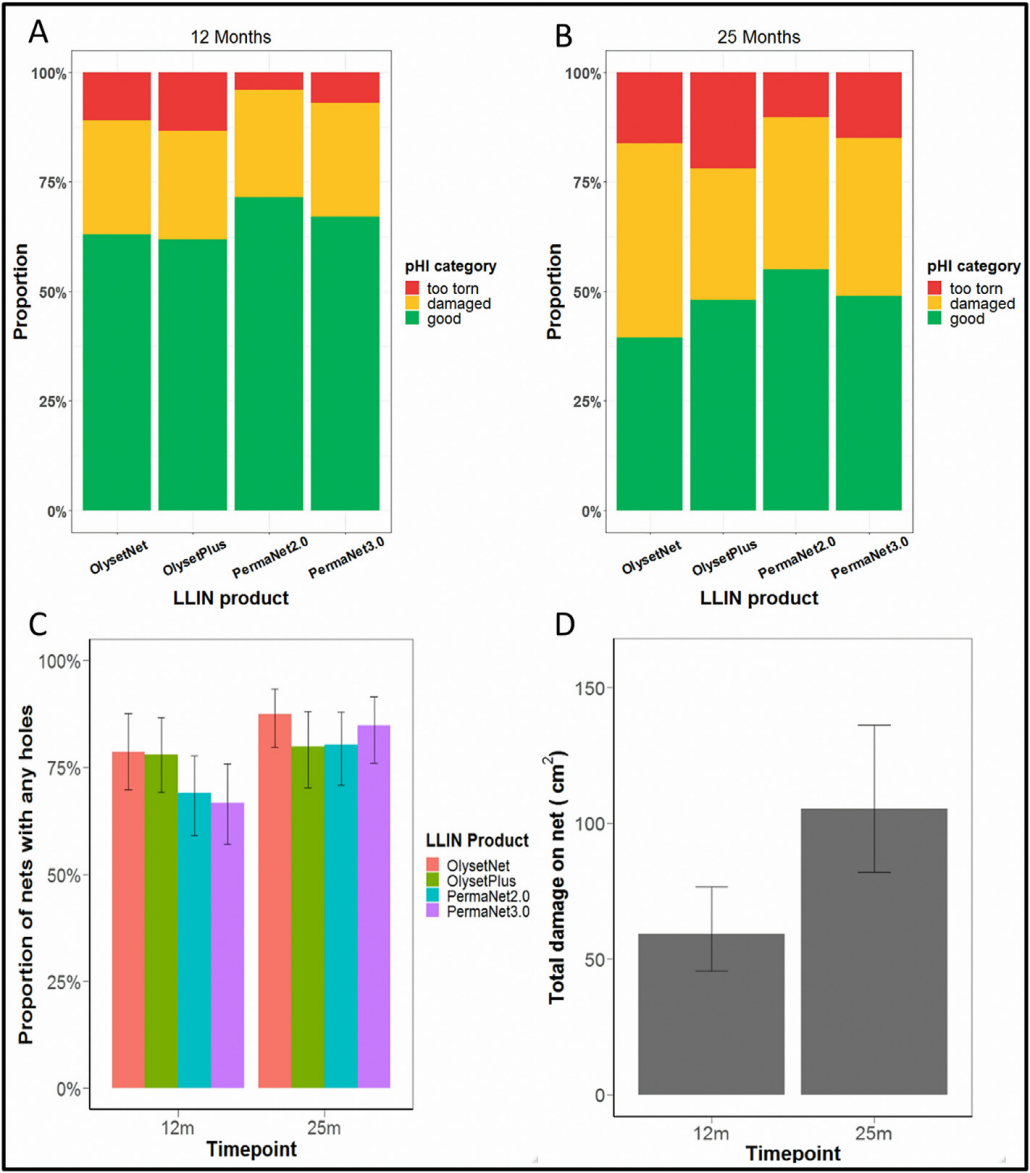
Physical integrity outcomes at 12 and 25 months post-distribution. **A** Percentage of collected nets in each pHI category (‘too torn’, ‘damaged’, ‘good’) at 12 months. **B** Percentage of collected nets in each pHI category at 25 months. **C** Percentage of nets with at least one hole. **D** Mean total surface area of damage per net at 12 and 25 months post-distribution across all LLIN products.

At 12 months post-distribution, the proportion of nets classified as ‘too torn’ on the pHI scale was 0.066 (Fig. 1A), with this proportion approximately doubling after 25 months (Fig. 1B) to 0.125 (OR: 2.017, 95% CI: 1.268–3.208, *P* < 0.001; Supplementary Table S2). There was no significant difference in the proportion of nets that were ‘too torn’ between LLIN products (*P* = 0.644).

When categorised by the type of housing they were collected from, it was observed that nets from traditional housing were more likely to be in poor physical condition than those from improved housing (OR: 3.350, 95% CI: 1.865–6.016, *P* = 0.003; Supplementary Table S2). After 25 months in operational use, the proportion of nets from traditional housing categorised as ‘too torn’ was 0.297 compared to 0.112 for improved housing (Supplementary Fig. S2).

#### 3.1.2 Proportion of nets with at least one hole

The proportion of nets of each type with at least one hole at 12- and 25 months post-distribution is shown in Fig. 1C. The overall proportion of nets with at least one hole after 12 months in operational conditions was 0.727, increasing to 0.829 after 25 months (OR: 1.821, 95% CI: 1.289–2.571, *P* < 0.001). There was no difference in the proportion of nets with at least one hole between the four LLIN products tested at any time-point (*P* = 0.306).

#### 3.1.3 Total surface area of holes

There was no difference in total hole area between any of the four LLIN products tested (*P* = 0.270). However, across all net types there was an overall increase in holed area from 12 months post-distribution to 25 months post-distribution (*P* = 0.0005; Fig. 1D), which approximately doubled from 59.33 cm^2^ (95% CI: 45.08–78.25) to 105.49 cm^2^ (95% CI: 83.43–136.86).

### 3.2 Chemical integrity

At baseline, all net samples tested met or exceeded the minimum target dose of active ingredients per their respective manufacturer specifications (Table 2).

**Table 2:** Mean chemical content (in g/kg) for each active ingredient in each LLIN product at baseline, 12 months, and 25 months post-distribution. Values in parentheses indicate 95% confidence interval

Active ingredient	LLIN product	Time-point		
		Baseline	12 months	25 months
Deltamethrin	PermaNet 2.0	1.3 (0.8–1.9)	1.1 (0.9–1.3)	0.7 (0.5–0.9)
	PermaNet 3.0	5.0 (4.1–5.9)	4.2 (4.0–4.5)	3.5 (3.2–3.8)
Permethrin	Olyset Net	19.5 (19.9–21.1)	17.0 (16.4–17.6)	18.2 (17.6–18.7)
	Olyset Plus	16.1 (13.6–18.5)	14.5 (13.7–15.4)	17.4 (16.5–18.3)
PBO	PermaNet 3.0	26.8 (22.9–30.7)	15.3 (13.7–16.9)	11.0 (9.4–12.7)
	Olyset Plus	8.2 (6.7–9.8)	5.0 (4.4–5.7)	3.7 (3.0–4.3)

#### 3.2.1 Deltamethrin

**Fig. 2:**
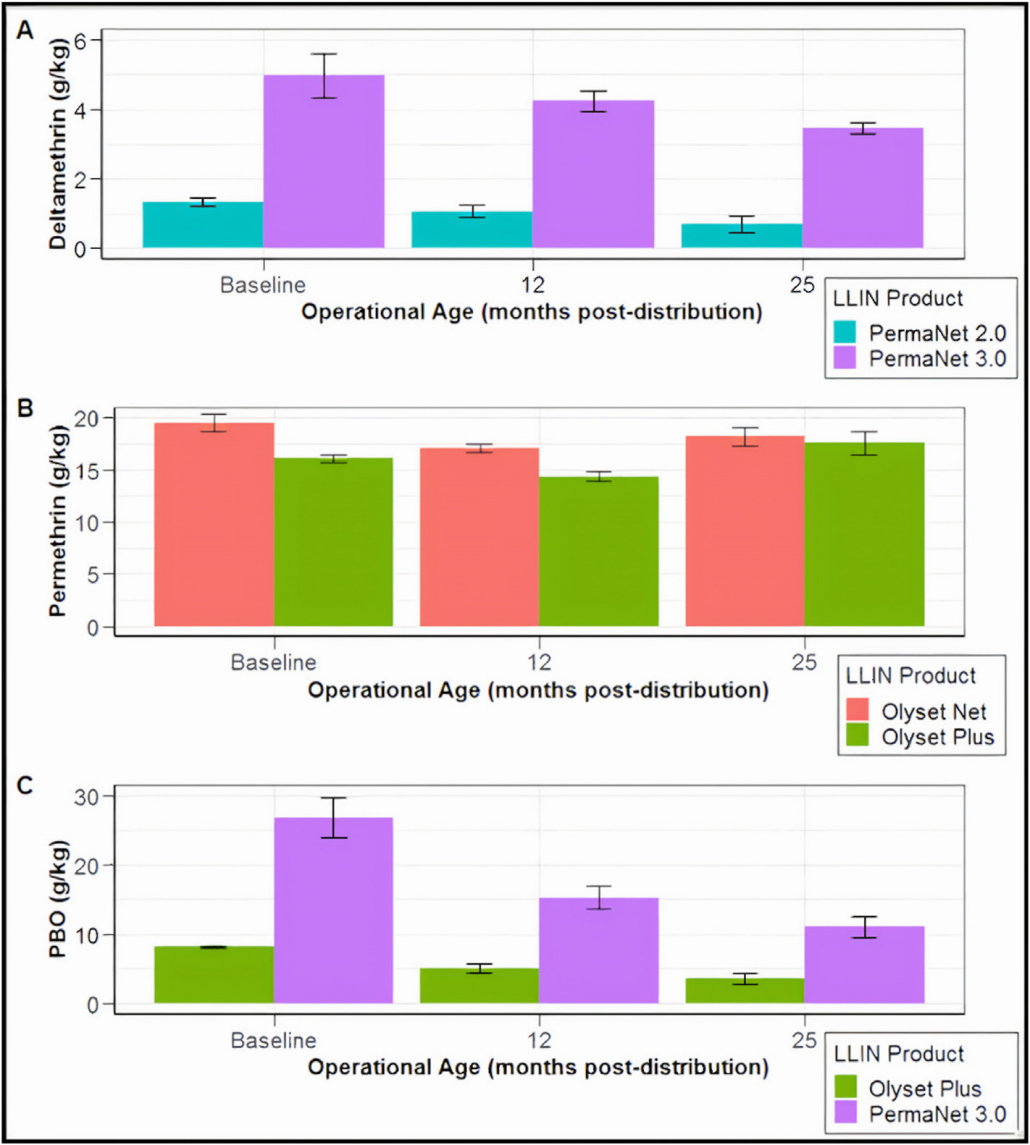
Mean concentration of deltamethrin (**A**), permethrin (**B**) and PBO (**C**) detected in net samples at each sampled time-point (measured using HPLC). Error bars indicate 95% confidence intervals.

The deltamethrin content of PermaNet 3.0 was lower at each subsequent time-point (*P* ≤ 0.001; Fig. 2A). In the period from baseline to 25 months, mean deltamethrin content of PermaNet 3.0 nets declined from 4.98 g/kg (95% CI: 4.08–6.01) to 3.48 g/kg (95% CI: 3.19–3.78). Despite this, the deltamethrin content of all PermaNet 3.0 nets collected at 25 months remained within the range of the target dose (3.0–5.0 g/kg). For PermaNet 2.0, mean deltamethrin content after 25 months was not statistically different from baseline (*P* = 0.071).

#### 3.2.2 Permethrin

The permethrin content of Olyset Plus varied across the sampled time-points (*P* < 0.001; Fig. 2B) however pairwise comparison indicated no overall difference between baseline and the final time-point at 25 months (*P* = 0.591). Mean permethrin content in Olyset Plus at baseline was 16.08 (95% CI: 13.70–18.62), declining to 14.54 (95% CI: 13.64–15.35) after 12 months, then increasing to 17.39 (95% CI: 16.53–18.22) after 25 months. A similar pattern was observed for Olyset Net, with permethrin content varying across time-points overall (*P* < 0.001), yet pairwise comparison indicating no overall difference between baseline and the 25-month time-point (*P* = 0.327).

#### 3.2.3 PBO

The PBO content of PermaNet 3.0 declined across the sampled time-points (*P* < 0.001; Fig. 2C). PBO content for PermaNet 3.0 at baseline was 26.81 g/kg (95% CI: 22.80–31.07) before declining sharply to 15.28 g/kg (95% CI: 13.74–16.71) after 12 months (*P* = 0.001), then falling further to 11.03 g/kg (95% CI: 9.35–12.67) after 25 months (*P* = 0.001).

A similar downwards trend in PBO was observed for Olyset Plus across time-points (*P* < 0.001). At baseline mean PBO content was 8.17 g/kg (95% CI: 6.51–9.82) before declining to 5.03 g/kg (95% CI: 4.37–5.74) after 12 months (*P* = 0.002). From 12 months to 25 months post-distribution, PBO content further fell to 3.66 g/kg (95% CI: 2.97–4.28, *P* = 0.013).

### 3.3 Bioefficacy

#### 3.3.1 Cone bioassay: pyrethroid-susceptible *An. gambiae*

All LLINs were effective per WHO definition against the pyrethroid-susceptible ‘Kisumu’ strain (defined as achieving either 95% knockdown or 80% mortality), both when new and 12 months post-distribution. Overall mean cone mortality was 96.93% (95% CI: 95.77–98.10%) at baseline. Adjusted cone mortality was statistically indistinguishable between LLIN products (*P* = 0.522) and did not vary significantly between time-points (*P* = 0.589).

#### 3.3.2 Cone bioassay: pyrethroid-resistant *An. gambiae*

**Fig. 3:**
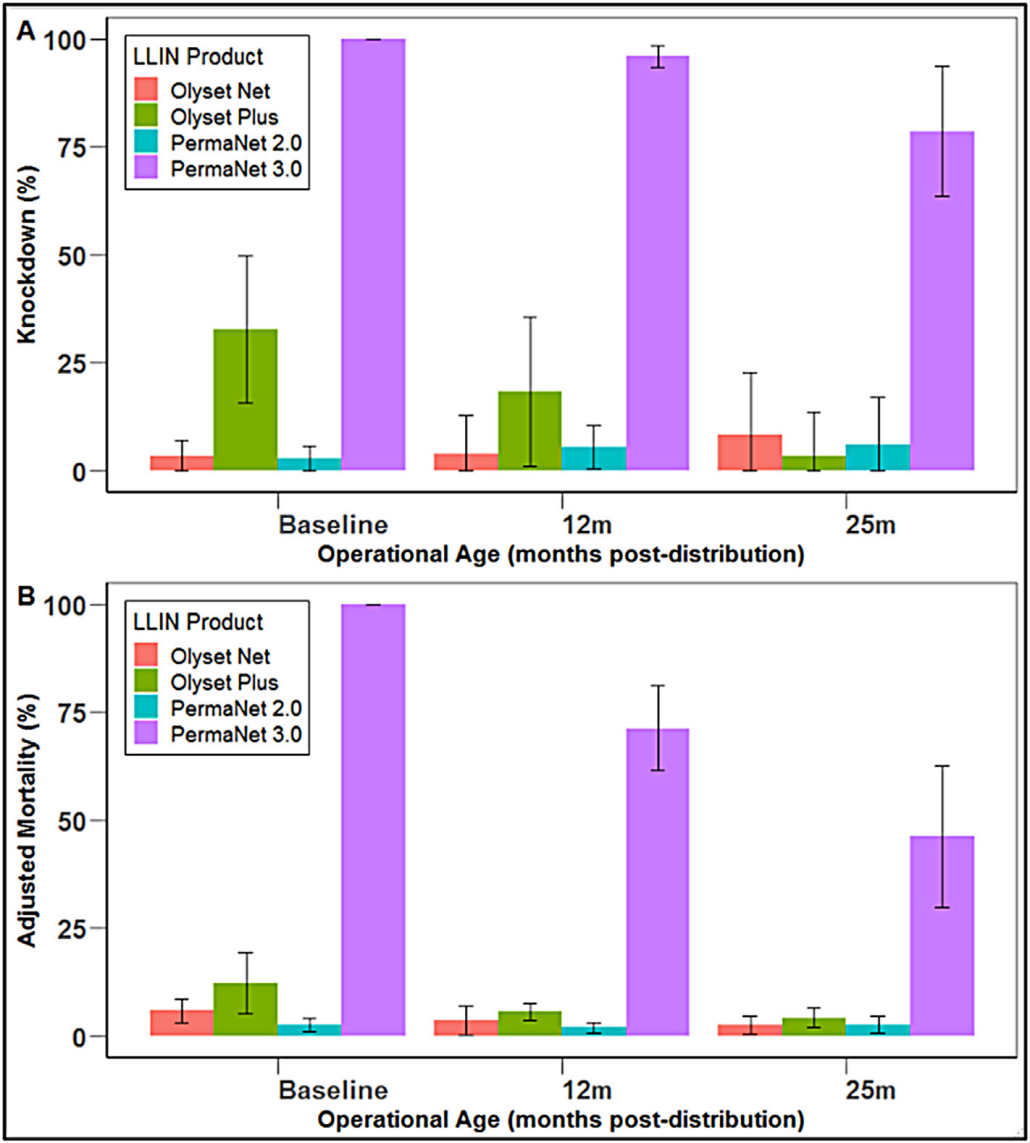
Mean knockdown (**A**) and adjusted mortality (**B**) in WHO cone bioassays with pyrethroid-resistant *An. gambiae* (*s.s.*) strain ‘Busia’ for each LLIN product tested at baseline, 12 months, and 25 months in the field.

Bioefficacy against the pyrethroid-resistant strain in cone assays varied between PBO-LLINs. Knockdown for PermaNet 3.0 remained very high throughout, achieving 99.7% (95% CI: 97.26–99.65; Fig. 3A) at baseline and remaining stable to 12 months (*P* = 0.441), though declining to 78.57% (95% CI: 63.57–93.58%, *P* < 0.001) after 25 months. PermaNet 3.0 was fully lethal against the pyrethroid-resistant strain when new, but mortality declined with operational use, falling by 26.8% (95% CI: 16.28–37.33%) for each year in the field (*P* < 0.001; Fig. 3B). In comparison, both mortality and knockdown with PermaNet 2.0 against the pyrethroid-resistant strain was very low at all time-points (3% and 6% respectively).

Knockdown with Olyset Plus was 46.98% (95% CI: 18.55–79.13%) when new but fell considerably to 3.54% (95% CI: 0.7–10.54%) after two years (*P* = 0.005). Mortality with Olyset Plus in cone assays was low throughout, killing 12.19% (95% CI: 5.45–17.01%) at baseline and 3.34% (95% CI: 0–8.71%) after two years but with no significant difference between time-points (*P* = 0.226; Fig. 3B). Knockdown and mortality with Olyset Net was low at all time-points (9% and 6% respectively).

#### 3.3.3 Wireball assay: pyrethroid-resistant *An. gambiae*

Due to the unexpectedly low bioefficacy of Olyset Plus in the WHO cone assay, the same net samples were assessed in WHO wireball assays. Olyset Net was also assessed in wireball assays for comparison.

**Fig. 4:**
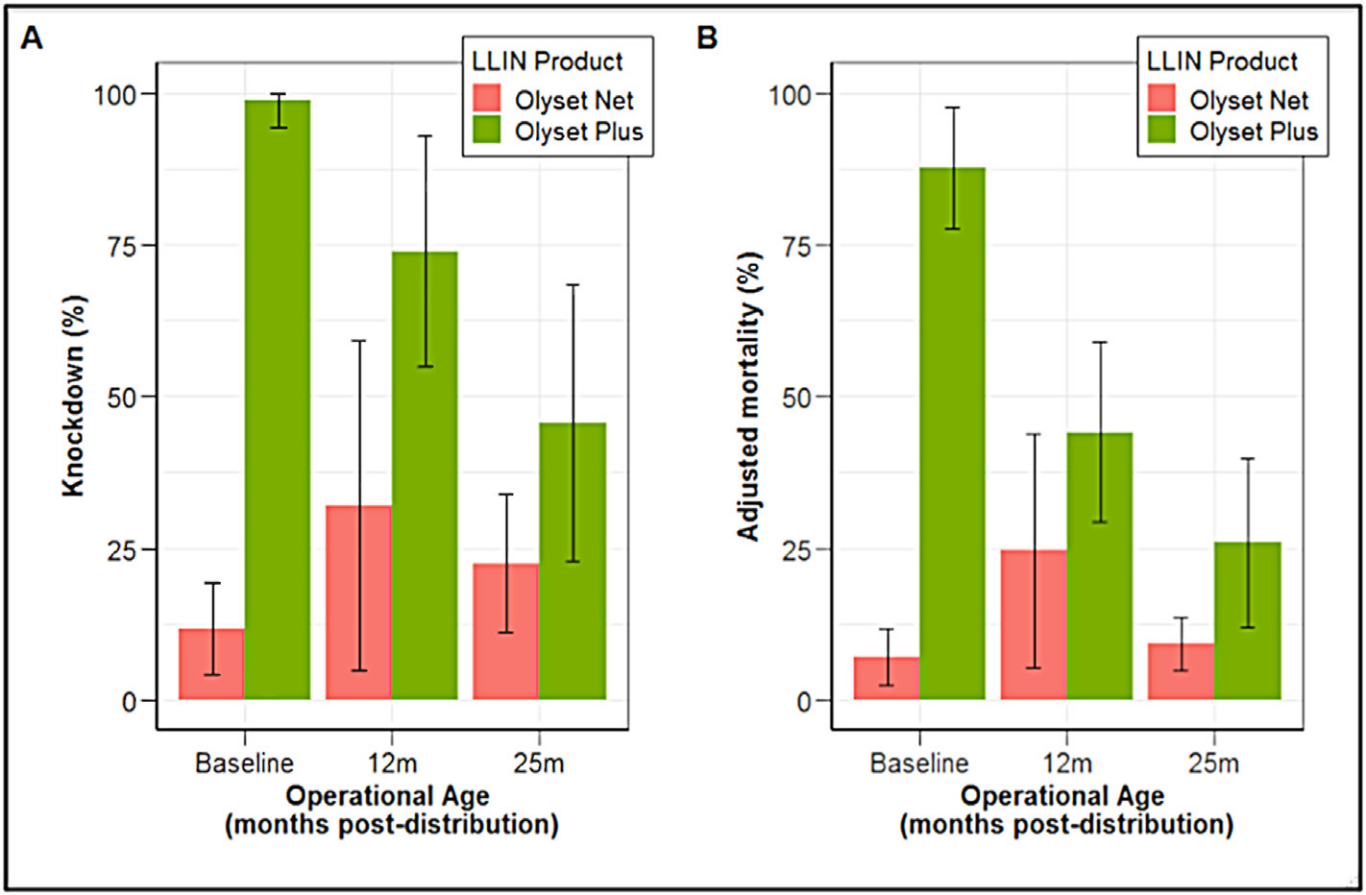
Mean knockdown (**A**) and adjusted mortality (**B**) in WHO wireball assays with pyrethroid-resistant *An. gambiae* strain ‘Busia’ for Olyset Net and Olyset Plus at baseline, 12 months, and 25 months in the field.

In the wireball assay, Olyset Plus knocked down 98.93% (95% CI: 94.43–100%; Fig. 4A) of pyrethroid-resistant mosquitoes at baseline. After 12 months knockdown had not significantly reduced (73.92%, 95% CI: 54.88–92.97%, *P* = 0.376); however, there was an overall decline to 45.72% (95% CI: 22.84–68.62, *P* = 0.021) after 25 months. Mortality for Olyset Plus against the pyrethroid-resistant strain in wireball assays at baseline was similarly improved compared to the cone assay, killing 87.72% at baseline (95% CI: 77.68–97.76%; Fig. 4B). However, after 12 months mortality has declined to 44.15% (95% CI: 29.32–58.98%, *P* = 0.002) though the subsequent decline to 25.92% (95% CI: 11.92–39.93%) at 25 months was not statistically significant (*P* = 0.216).

The bioefficacy of Olyset Net in the wireball assay was low at all sampled time-points, with overall mean knockdown and mortality 22% and 13.5% respectively.

### 3.4 Relationship between chemical integrity and bioefficacy

**Fig. 5:**
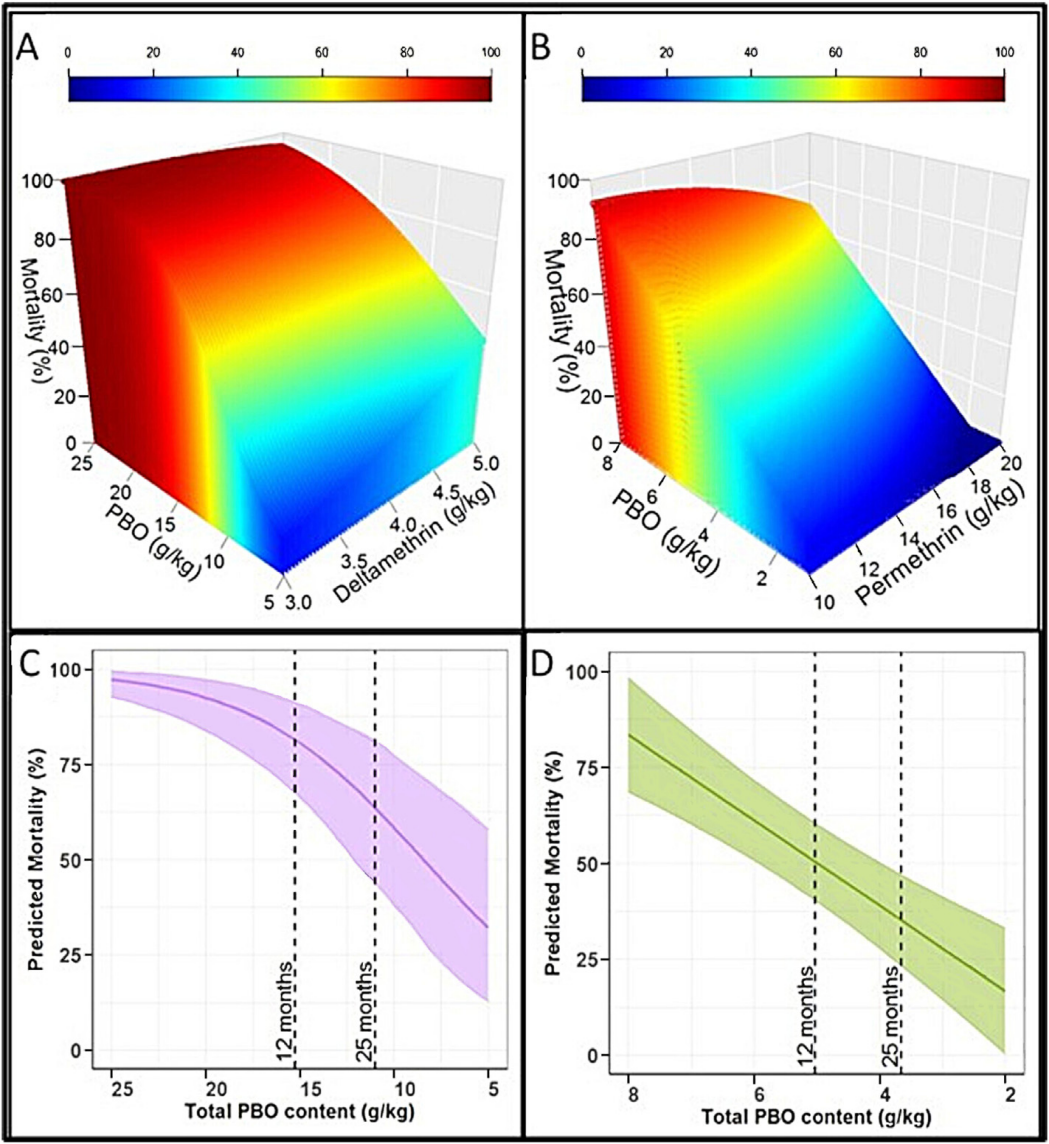
Relationship between total chemical content and bioefficacy against pyrethroid-resistant *An. gambiae* (*s.s*.). **A** PermaNet 3.0 in WHO cone bioassays. **B** Olyset Plus in WHO wireball bioassays. **C** PermaNet 3.0 in WHO cone with deltamethrin value fixed at mean (4.42 g/kg). **D** Olyset Plus in WHO wireball with permethrin value fixed at mean (15.45 g/kg).

The relationship between chemical integrity and predicted mortality for the pyrethroid-resistant *An. gambiae* (*s.s.*) ‘Busia’ line is shown in Fig. 5. For PermaNet 3.0 in the WHO cone bioassay, mortality was dependent on both total deltamethrin content and total PBO content, as indicated by a significant interaction between the two variables (*P* < 0.001; Fig. 5A). Modelling indicated there is a non-linear association between PBO content and mortality, with mortality falling more sharply with each consecutive g/kg of PBO that is lost (Fig. 5C). When the deltamethrin value was fixed at the mean of the data (4.42 g/kg), a reduction in PBO from 25 g/kg to 15 g/kg resulted in predicted mortality falling from 98% to 90%. Furthermore, a reduction in PBO content from 15 g/kg to 5 g/kg resulted in a decline in predicted mortality from 90% to 57%. Consequently, the model predicted that to achieve 80% mortality against this pyrethroid-resistant mosquito strain a minimum of 11 g/kg PBO was needed.

For Olyset Plus in the WHO wireball bioassay, mortality had no statistical relationship with total permethrin content (*P* = 0.583) and was instead directly correlated with total PBO content (*P* < 0.001; Fig. 5B). Modelling indicated there was a linear association between PBO content and predicted mortality, with mortality falling by 11.12% for each g/kg PBO that is lost (Fig. 5D). The model predicted that to achieve 80% mortality against this strain, a minimum of 7.7 g/kg PBO was needed.

## 4 Discussion

### 4.1 Physical integrity

There was no difference in physical integrity outcomes between any of the four LLINs tested after 25 months in operational conditions. Thus, PBO-LLINs nets were as physically durable as their pyrethroid-only equivalents. Furthermore, it was observed that nets sampled from ‘traditional’ thatched-roof housing were almost three times more likely to be in the most severely damaged category than nets from ‘improved’ iron-roofed housing. While this disparity may be associated with the housing structure itself (such as the presence of straw), housing type may in fact be an indicator of other household variables such as the construction of the bed frame, the presence of animals indoors, or the type of cooking material used in the household (Gnanguenon et al., 2014). More generally, these household variables are expected to be indicative of overall socioeconomic status which may impact an individualʼs day-to-day behaviour and use of their net. Nonetheless, there may be an argument to distribute nets more frequently than three years in regions where traditional housing remains common. It should be noted that the net attrition rate was high, with adequate coverage of LLINs (one LLIN for every two residents) decreasing from 71% at baseline to 35% after 25 months (Maiteki-Sebuguzi et al., unpublished data), indicating that LLIN attrition after distribution is an issue. If, as might be expected, individuals chose to discard damaged nets at a higher rate than nets in good condition, then the physical damage observed in the present study may be an underestimate.

The current physical integrity outputs outlined in the WHO durability guidelines cannot be directly interpreted in terms of personal and community protection from mosquito bites. There is a need to better understand the impact of declining physical integrity on both mosquito blood-feeding inhibition and mortality. There is empirical evidence that damage to pyrethroid LLINs reduces personal protection from bites, but that mortality is independent from holed surface area and instead dependent on resistance status (Randriamaherijaona et al., 2015). Consequently, damaged LLINs would be expected to retain community effect against mosquito populations that are susceptible to their chemistry. Despite this, the median retention time of LLINs is well below three years in many settings (1.64 years across sub-Saharan Africa and 1.66 years for Uganda) (Bertozzi-Villa et al., 2021). Given evidence that perception of physical integrity is the primary consideration in retention (Koenker et al., 2014), developing more durable LLIN products may have epidemiological impacts beyond what would be indicated by studies of mosquito behaviour, due to improved retention.

In the current WHO durability guidelines, the location of holes on the net surface is not factored into categorisation of net condition by proportionate Hole Index. Recent behavioural experiments demonstrate that *An. gambiae* host-seeking activity occurs primarily on the top surface of the LLIN (Lynd & McCall, 2013; Sutcliffe & Yin, 2014, 2021; Parker et al., 2015; Sutcliffe et al., 2017). This highlights an important knowledge gap in the relationship between hole location on a net and the probability of mosquito entry and net effectiveness.

### 4.2 Chemical integrity

The pyrethroid content of the LLINs assessed was relatively stable across the two years of the study, with the exception of PermaNet 3.0 which declined by ∼30% (yet was still within the manufacturerʼs target range). The stability of pyrethroids over two years observed here is consistent with studies from a range of settings (Lorenz et al., 2014, 2020; Toé et al., 2019). In contrast, the PBO content of both PBO-LLINs declined more rapidly over the same time period, with under half of the initial content remaining after 25 months. Nonetheless, despite this decline in PBO content, the concurrent trial of epidemiological outcomes in the study site demonstrated that PBO-LLINs maintained superior protection over their conventional equivalents up to 25 months (Staedke et al., 2020; Gleave et al., 2021).

While a strong correlation between total PBO content and bioefficacy was observed for both PBO-LLINS, this relationship may not be causal and total chemical content quantified by HPLC may not be representative of the concentration at the surface bioavailable to mosquitoes. There is currently a lack of tools for quantifying the concentration important for future studies seeking to link chemical composition to bioefficacy.

### 4.3 Bioefficacy

Both Olyset Plus and PermaNet 3.0 tested demonstrated superior bioefficacy against the pyrethroid-resistant strain than their pyrethroid-only equivalents. This observation is consistent with the previously reported finding that these nets reduced childhood parasitaemia in the study area where these nets were collected (Staedke et al., 2020). However, while both PBO-LLINs tested were highly effective against the pyrethroid-resistant strain at baseline, their bioefficacy diminished with operational use (with the mortality associated with Olyset Plus and PermaNet 3.0 decreasing to 26% and 46%, respectively, after two years). The diminishing differential in bioefficacy between PBO-LLINs and their pyrethroid-only equivalents is also consistent with the observation that differential impact on childhood parasitaemia narrowed over the same time. The steep reduction in bioefficacy with both PBO-LLINs against a study site-specific pyrethroid-resistant strain is greatly concerning. These nets were distributed with the expectation they will be replaced after three years, yet these findings indicate that they have greatly diminished killing effect after the first two years. While the bioefficacy values themselves are specific to the ‘Busia’ strain, there is an urgent need to investigate if this downwards trend is observed in other settings. Given these findings, there is an argument that, within the Ugandan context, LLINs should be distributed on a two-rather than three-year cycle to maintain efficacy.

The low knockdown and mortality observed with Olyset Plus in the WHO cone bioassay was in strong contrast with the high bioefficacy observed with the same nets in the WHO wireball bioassay. This difference in outcomes between methodologies may be associated with the excitorepellency of permethrin, manifesting as reduced contact with the net surface. As the wireball method surrounds the mosquito on all sides with netting, there is no insecticide-free surface to rest on and a greater insecticidal effect is observed. Consequently, future investigations with excito-repellent LLINs may wish to also include an assay that prevents avoidance from the net, such as the WHO wire-ball assay (WHO, 2006). The WHO tunnel test would also address excito-repellency; however, in practice the aforementioned ethical issues prevent many institutes from performing it.

## 5 Conclusions

This LLIN durability study was conducted alongside a trial into the epidemiological effectiveness of PBO-LLINs in protecting against the bites of *Anopheles* mosquitoes in Uganda, where there is widespread pyrethroid resistance. Here, we demonstrate that both Olyset Plus and PermaNet 3.0 were as physically durable as their conventional equivalents and had superior bioefficacy against pyrethroid-resistant *An. gambiae* (*s.s.*) mosquitoes from the trial site. However, the superiority of PBO-LLINs over conventional LLINs in bioassays narrowed with the operational life of the net, correlating with a decline in PBO content. Additionally, we observed that nets collected from traditional thatched-roof housing were far more likely to be severely damaged than nets from improved iron-roofed housing. The diminished bioefficacy of PBO-LLINs against pyrethroid-resistant mosquitoes after just two years of operational use is of great concern and there is an urgent need to assess the durability of these LLIN products in other settings. Given these findings, we suggest that control programmes should consider distributing PBO-LLINs at more frequent intervals than three years and prioritise regions where traditional housing is common. Additionally, the contrasting performance of the same Olyset Plus nets in the WHO cone assay and the WHO wireball bioassay highlights that LLIN products with excito-repellent properties should be assessed with approaches that minimise avoidance from the net surface.

## Funding

This project was funded primarily by the Against Malaria Foundation, with additional funding from the 10.13039/501100002992Department for International Development, the Innovative Vector Control Consortium, the 10.13039/100000865Bill and Melinda Gates Foundation. The content of the manuscript is solely the responsibility of the authors.

## Ethical approval

The trial was approved by the 10.13039/501100017230Uganda National Council for Science and Technology (UNCST Ref. HS 2176), Makerere University School of Medicine Research & Ethics Committee (SOMREC 2016-133), London School of Hygiene & Tropical Medicine Ethics Committee (LSHTM Ref. 12019), and the Liverpool School of Tropical Medicine (LSTM Ref. 16-072), which is the sponsoring institute.

## CRediT author statement

Frank Mechan: Investigation, methodology, formal analysis, investigation, resources, data curation, visualization, writing - original draft. Agaba Katureebe: Investigation, methodology, supervision, project administration, data curation, resources, writing - original draft. Violet Tuhaise: Data curation, investigation, resources, supervison, writing - review & editing. Martin Mugote: Investigation, resources, writing - review & editing. Ambrose Oruni: Investigation, resources, writing - review & editing. Ismail Onyige: Investigation, resources, writing - review & editing. Kawesa Bumali: Investigation, resources, writing - review & editing. Jonathan Thornton: Investigation, resources, methodology, writing - review & editing. Kilama Maxwell: Investigation, methodology, writing - review & editing. Mary Kyohere: Investigation, methodology, supervision, project administration, data curation, resources, writing - review & editing. Moses R. Kamya: Project administration, methodology, investigation, data curation, writing - review & editing. Peter Mutungi: Data curation, investigation, resources, writing - review & editing. Simon P. Kigozi: Methodology, formal analysis, data curation, writing - review & editing. Adoke Yeka: Methodology, investigation, resources, data curation, project administration, writing - review & editing. Jimmy Opigo: Conceptualization, methodology, resources, writing - review & editing. Catherine Maiteki-Sebuguzi: Project administration, resources, data curation, writing - review & editing. Samuel Gonahasa: Conceptualization, supervision, methodology, project administration, writing - review & editing. Janet Hemingway: Conceptualization, supervision, methodology, writing - review & editing. Grant Dorsey: Conceptualization, formal analysis, methodology, writing - review & editing. Lisa J. Reimer: Supervision, methodology, writing - review & editing. Sarah G. Staedke: Conceptualization, supervision, methodology, project administration, writing - review & editing. Martin J. Donnelly: Conceptualization, supervision, methodology, writing - original draft. Amy Lynd: Conceptualization, supervision, investigation, writing - original draft, methodology, formal analysis, investigation, resources, data curation, visualization. All authors read and approved the final manuscript.

## Declaration of competing interests

The authors declare that they have no known competing financial interests or personal relationships that could have appeared to influence the work reported in this paper.
